# The Inhibition of MicroRNA-139-5p Promoted Osteoporosis of Bone Marrow-Derived Mesenchymal Stem Cells by Targeting Wnt/Beta- Catenin Signaling Pathway by NOTCH1

**DOI:** 10.4014/jmb.1908.08036

**Published:** 2019-11-18

**Authors:** Yimiao Feng, Pengbo Wan, Linling Yin, Xintian Lou

**Affiliations:** 1 Department of Orthodontics, Second Affiliated Hospital, College of Medicine, Zhejiang University, Hangzhou, Zhejiang Province 30009, P.R. China; 2 Shangqiu Medical College, Shangqiu, Henan Province 476100, P.R. China; 3 Department of Stomatology, Shanghai General Hospital, Shanghai Jiao Tong University School of Medicine (originally named “Shanghai First People’s Hospital”) Shanghai 200080, P.R. China; 4 Department of Dentistry, Punan Hospital of Pudong New District, Shanghai 200125, P.R. China

**Keywords:** MicroRNA-139-5p, osteogenic differentiation, bone marrow-derived mesenchymal stem cell, Wnt, NOTCH1, β-catenin

## Abstract

We investigated the therapeutic effects of microRNA-139-5p in relation to osteoporosis of bone marrow-derived mesenchymal stem cell (BMSCs) and its underlying mechanisms. In this study we used a dexamethasone-induced in vivo model of osteoporosis and BMSCs were used for the in vitro model. Real-time quantitative polymerase chain reaction (RT-PCR) and gene chip were used to analyze the expression of microRNA-139-5p. In an osteoporosis rat model, the expression of microRNA-139-5p was increased, compared with normal group. Downregulation of microRNA-139-5p promotes cell proliferation and osteogenic differentiation in BMSCs. Especially, up-regulation of microRNA-139-5p reduced cell proliferation and osteogenic differentiation in BMSCs. Overexpression of miR-139-5p induced Wnt/β-catenin and down-regulated NOTCH1 signaling in BMSCs. Down-regulation of miR-139-5p suppressed Wnt/β-catenin and induced NOTCH1 signaling in BMSCs. The inhibition of NOTCH1 reduced the effects of anti-miR-139-5p on cell proliferation and osteogenic differentiation in BMSCs. Activation of Wnt/β-catenin also inhibited the effects of anti-miR-139-5p on cell proliferation and osteogenic differentiation in BMSCs. Taken together, our results suggested that the inhibition of microRNA-139-5p promotes osteogenic differentiation of BMSCs via targeting Wnt/β-catenin signaling pathway by NOTCH1.

## Introduction

Bone tissue engineering (BTE) brings new opportunities for the repair of bone defects and bone reconstruction [1]. Due to the increasingly in-depth research on bone marrow-derived mesenchymal stem cells (BMSCs) in recent years, they have come to serve as the preferred seed cells; and how to promote their osteogenic differentiation and proliferation more effectively is of key importance in BTE [2, 3]. The effects of kidney-tonifying traditional Chinese medicine (TCM) on promoting the osteogenic differentiation of BMSCs have been extensively acknowledged based on the Kidney Governing Bone theory in TCM, which has provided new thinking in the field of BTE [2].

As a type of key seed cell in osteogenic differentiation as well as in bone development and reconstruction, stem cells are regulated by classic epigenetics. In addition, it is also being discovered through more and more studies that miRNA plays an important regulatory role in osteogenic differentiation [4, 5]. As a class of endogenous non-coding RNA, miRNA can inhibit translation while it promotes degradation through specific binding with the target gene mRNA, and thus plays a post-transcriptional regulation role in gene expression [6]. miRNA plays an important regulatory role in the proliferation regulation of all kinds of cells, while also maintaining differentiation. The regulatory mechanism of miRNA on the osteogenic differentiation of stem cells has become a hot topic in bone tissue engineering in recent years [4].

The Notch gene was first discovered in fruit fly, which was so named since its loss of some function led to the notch in the fruit fly wing margin. The Notch signaling pathway exists extensively in multiple vertebrate and invertebrate species, and participates in regulating the vital activities in a series of physiological and pathological processes, including cell fate determination, differentiation, proliferation, apoptosis, epithelial-mesenchymal transition (EMT), angiogenesis, migration and adhesion. As is shown in relevant research, the Notch signaling pathway plays different roles in the differentiation of different stem cells, and different Notches have different effects even in the differentiation of the same stem cell.

The Wnt protein family is a group of secretory proteins that are involved in regulating numerous embryonic development processes [7]. The classical Wnt/β-catenin signaling pathway also plays an important role in skeletal development and osteogenic differentiation. The classical Wnt signaling pathway regulates the β-catenin protein level [8]. It is confirmed in research that the classical Wnt/β-catenin signaling pathway promotes the osteogenic differentiation of stem cells [9]. Classical Wnt signaling can promote the osteogenic differentiation of mesenchymal stem cells through the Wnt/β-catenin pathway, while inhibiting their differentiation into adipocytes [10]. Recent research discovered that interfering with Wnt/β-catenin signaling will inhibit the differentiation of osteogenesis precursor cells into osteoblasts, and convert them to adipocytes [11]. In the present study, we investigated whether the therapeutic effects of microRNA-139-5p promote osteogenic differentiation of bone marrow-derived mesen-chymal stem cells (BMSCs) and its underlying mechanisms.

## Materials and Methods

### Animal Experiments

The animal protocol was approved by the Committee on the Ethics of Animal Experiments of Second Affiliated Hospital of Zhejiang University. C57BL/6 mice (six-week-old male, 18-20 g) were purchased from Beijing Vital River Laboratory Animal Technology Co., Ltd. (China) and were given free access to food and water. They were caged individually under 22-24°C and 55-60% humidity with a 12 h light cycle. The present study used a dexamethasone-induced model of osteoporosis, as described previously [12]. Mice were divided into two groups: the sham and model groups.

### Culture of BMSCs

Male C57BL/6 mice (six-week-old male, 18-20 g) were sacrificed by cervical dislocation under anesthetization and disinfected with 75% alcohol for 3-5 min. The femurs and tibiae were carefully isolated and flushed with Dulbecco’s modified Eagle’s medium (DMEM; Gibco, USA). Cells were centrifugated at 300 ×*g* for 10 min, and re-suspended using red blood cell (RBC) lysis buffer (500 μl, Beyotime, China) for 10 min. Cells were centrifugated at 300 ×*g* for 10 min, and were re-suspended using PBS. Cells were re-suspended in DMEM supplemented with 10% fetal bovine serum (FBS; Gibco) in culture flasks at a density of 1 × 10^5^ cells/ml. Adherent cells were cultured by changing the medium every 3 days and the cells were used for subsequent experiments following 3 passages.

### Lentivirus-Mediated Cell Transfection

BMSCs were seeded into a 6-well plate and cultured overnight to reach 70% confluence. LV-miR-139-5p-overexpressing, si-NOTCH1 and LV-NC (negative control) were purchased from Hanbio (Shanghai, China). Subsequently, cells were transfected with different lentiviruses in the presence of ploybrene (8 μg/ml; Sigma-Aldrich, USA) for 24 h.

### Cell Viability Assay

After lentivirus-mediated cell transfection for 24, h, cells were seeded in 96-well culture plates at a density of 0.5 × 10^3^ cells/well for 48 h. MTT assay (20 μl, Invitrogen) was added into wells and cultured for 4 h at 37°C. Medium was replaced, and DMSO assay was added into wells and cultured for 20 min at 37°C. Absorbance was measured at 490 nm using a microplate reader (model 550, Bio-Rad, USA).

### Real-Time Quantitative Polymerase Chain Reaction (RT-PCR)

Total RNAs from cultured BMSCs were extracted using RNA Pure Rapid Extraction Kit (Bioteke Corporation, China). Total RNAs was amplified to complimentary DNA (cDNA) using a high-capacity cDNA reverse-transcription kit (Applied Biosystems, USA). RT-PCR was quantitatively determined using the SYBR Green Dye system (SYBR Premix Ex Taq (Tli RNase Plus) by an Applied Biosystems StepOnePlus real-time PCR System (USA). RT-PCR was as follows: at 95°C for 10 min, then 40 cycles consisting of 95°C for 15 sec, 60°C for 30 sec, and 72°C for 1 min, followed by 5 min incubation at 4°C.

### Dual Luciferase Reporter Assay

The 3'UTR seed region of Notch1 was synthesized and cloned into a pMIR-REPORT luciferase vector (Applied Biosystems; Thermo Fisher Scientifc, Inc.). The Notch1 construct or control construct was co-transfected into C BMSCs with miR-139 using Lipofectamine 2000 (Invitrogen; Thermo Fisher Scientifc, Inc.). After 48 h of transfection, luciferase activity was measured using the Dual Luciferase Reporter Assay system (Promega Corporation, USA).

### Western Blot Analysis

BMSCs were lysed in RIPA buffer (Beyotime, China) supplemented with PMSF (Beyotime) and total proteins were quantitatively determined using a BCA Protein Assay Kit (Beyotime). Equal total proteins were separated by 8-10% SDS-PAGE and transferred onto PVDF membranes (Millipore, USA). Membranes were separated by 10% SDS-PAGE and transferred onto PVDF membranes (Millipore). Membranes were blocked with 5% non-fat milk in TBST for 1 h, and then incubated overnight at 4°C with primary antibody against: Wnt, β-catenin, NOTCH1 and GAPDH. After washing with TBST, the blots were incubated with HRP-conjugated rabbit anti-goat IgGs at 37°C for 1 h and then visualized using enhanced chemiluminescence reagents.

### Immunofluorescence

Cells were washed with PBS and fixed with 4% paraformaldehyde for 15 min. Next, cells were blocked for 30 min using 0.25% TrisX-100 and incubated with NOTCH1 for 1 h at room temperature. After washing with PBS, cells were incubated with goat-555 secondary anti-body for 1 h and stained with DAPI for 15 min. After washing with PBS, cells were examined under a fluorescent microscope (Nikon Eclipse TE2000-S; Nikon, Japan).

### Statistical Analysis

All experimental data were presented as the means ± SD. The significant differences between the groups were analyzed by Student’s t-test or one-way analysis of variance (ANOVA) followed by Newman-Keul’s test. Differences were considered to be significant at *p* ≤ 0.05.

## Results

### Expression of MicroRNA-139-5p in Model of Osteoporosis

**Fig. 1:**
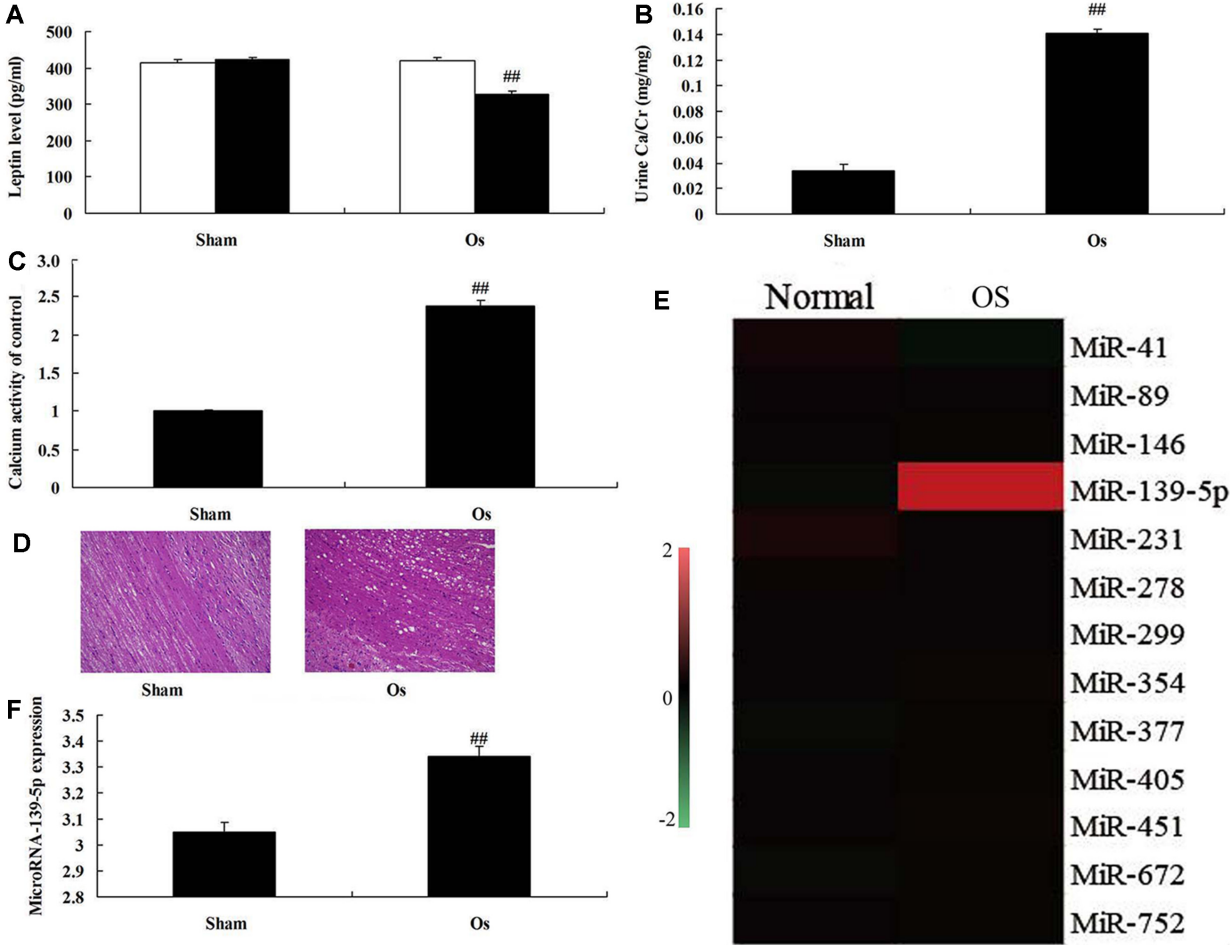
Expression of microRNA-139-5p in model of osteoporosis. Leptin levels (**A**), urine Ca/Cr (**B**), calcium activity levels (**C**), HE staining (**D**), gene chip (**E**), and QPCR (**F**) for microRNA-139-5p expression in n model of osteoporosis. Sham, sham group; OS, osteoporosis model group. ##*p* < 0.01 compared with sham group.

First, we investigated the changes of microRNA-139-5p in a model of osteoporosis. As shown in Figs. 1A-1C, leptin levels were inhibited, and urine Ca/Cr and calcium activity levels were increased in the model of osteoporosis, compared with the sham group. The results of HE staining showed that there were increases of bone cavity in the model of osteoporosis, compared with the sham group (Fig. 1D). Therefore, the expression of microRNA-139-5p in the model of osteoporosis was promoted, compared with the sham group (Figs. 1E and 1F). These results showed that microRNA-139-5p may regulate bone cell apoptosis in osteoporosis.

### MicroRNA-139-5p Regulates Cell Proliferation and Osteogenic Differentiation in BMSCs

**Fig. 2:**
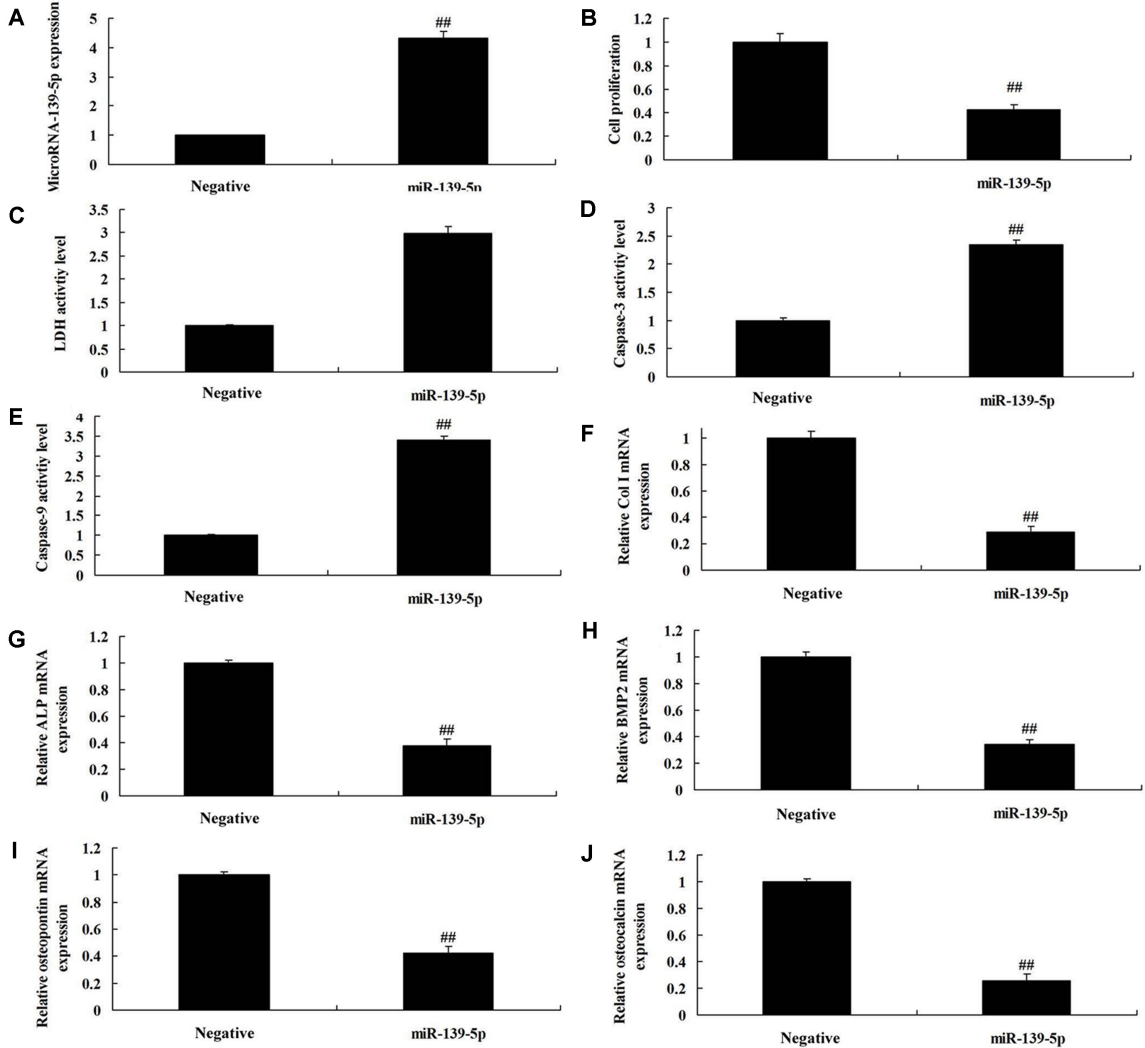
MicroRNA-139-5p regulates cell proliferation and osteogenic differentiation in BMSCs. MicroRNA-139-5p expression (**A**), cell proliferation (**B**), LDH activity level (**C**) and caspase-3/9 activity levels (D and E), and Col I (**F**), ALP (**G**), BMP2 (**H**), osteopontin (**I**), and osteocalcin (**J**) mRNA expressions. Negative, negative mimics group; miR-139-5p, over-expression of miR-139-5p group. ##*p* < 0.01 compared with negative mimics group.

**Fig. 3:**
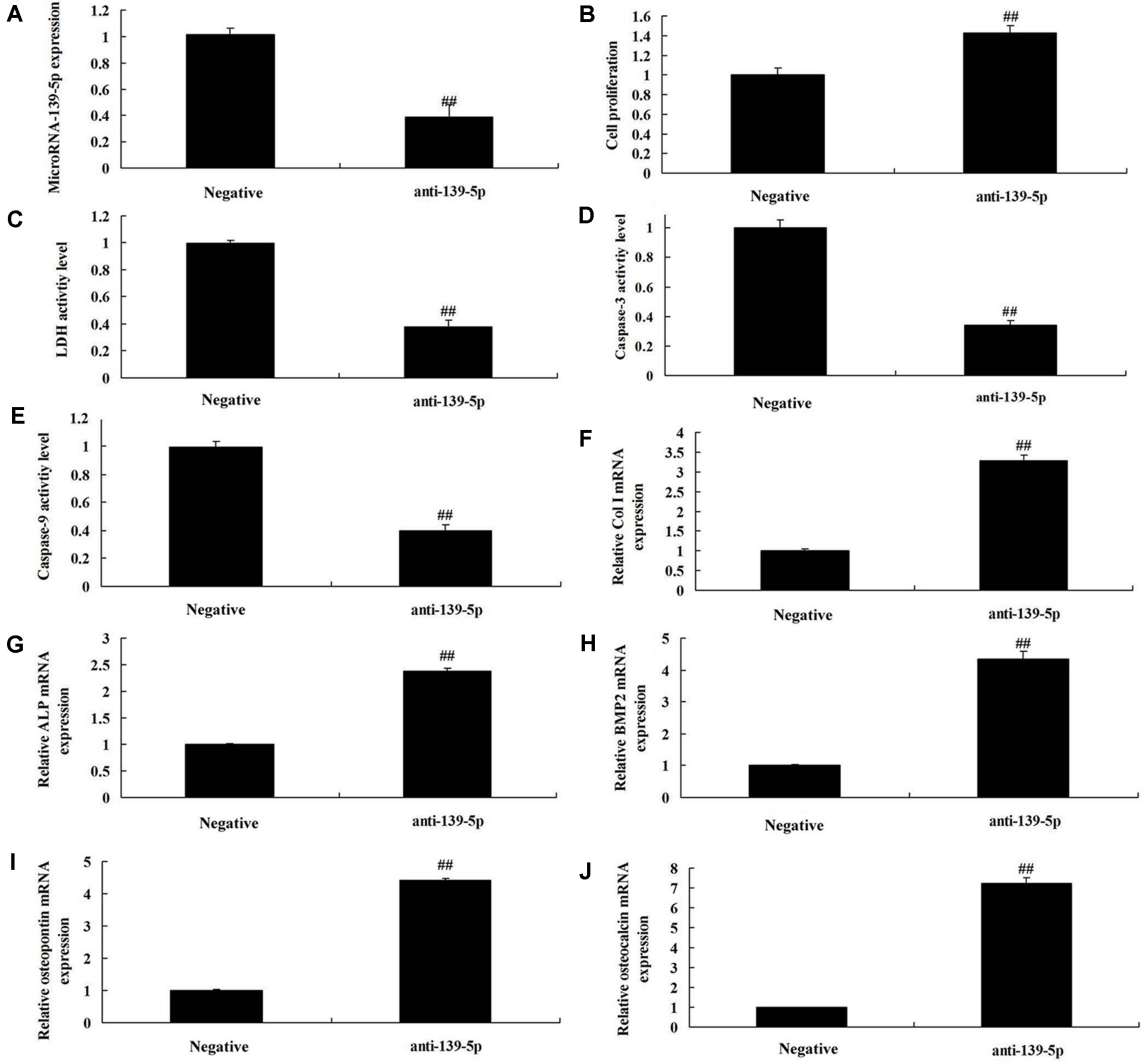
Anti-microRNA-139-5p regulates cell proliferation and osteogenic differentiation in BMSCs. MicroRNA-139-5p expression (**A**), cell proliferation (**B**), LDH activity level (**C**) and caspase-3/9 activity levels (D and E), and Col I (**F**), ALP (**G**), BMP2 (**H**), osteopontin (**I**), and osteocalcin (**J**) mRNA expressions. Negative, negative mimics group; anti-139-5p, down-regulation of miR-139-5p group. ##*p* < 0.01 compared with negative mimics group.

Then, we explored the mechanism of microRNA-139-5p on osteoporosis by up-regulating microRNA-139-5p expression using microRNA-139-5p mimics. There was an increase of microRNA-139-5p expression in BMSCs by over-expression of microRNA-139-5p, compared with the negative group (Fig. 2A). Over-expression of microRNA-139-5p reduced cell proliferation, increased LDH activity level and caspase-3/9 activity levels, and inhibited Col I, ALP, BMP2, osteopontin and osteocalcin mRNA expressions in BMSCs, compared with the negative group (Figs. 2B-2J). However, in this study we also used anti-microRNA-139-5p mimics to reduce the expression of microRNA-139-5p in BMSCs, compared with the negative group (Fig. 3A). Down-regulation of microRNA-139-5p increased cell proliferation, decreased LDH activity level and caspase-3/9 activity levels, and promoted Col I, ALP, BMP2, osteopontin and osteocalcin mRNA expressions in BMSCs, compared with the negative group (Figs. 3B-3J). So, microRNA-139-5p regulates cell proliferation and osteogenic differentiation in BMSCs to affect osteoporosis.

### MicroRNA-139-5p Regulates Wnt/β-Catenin Signaling Pathway by NOTCH1 in BMSCs

**Fig. 4:**
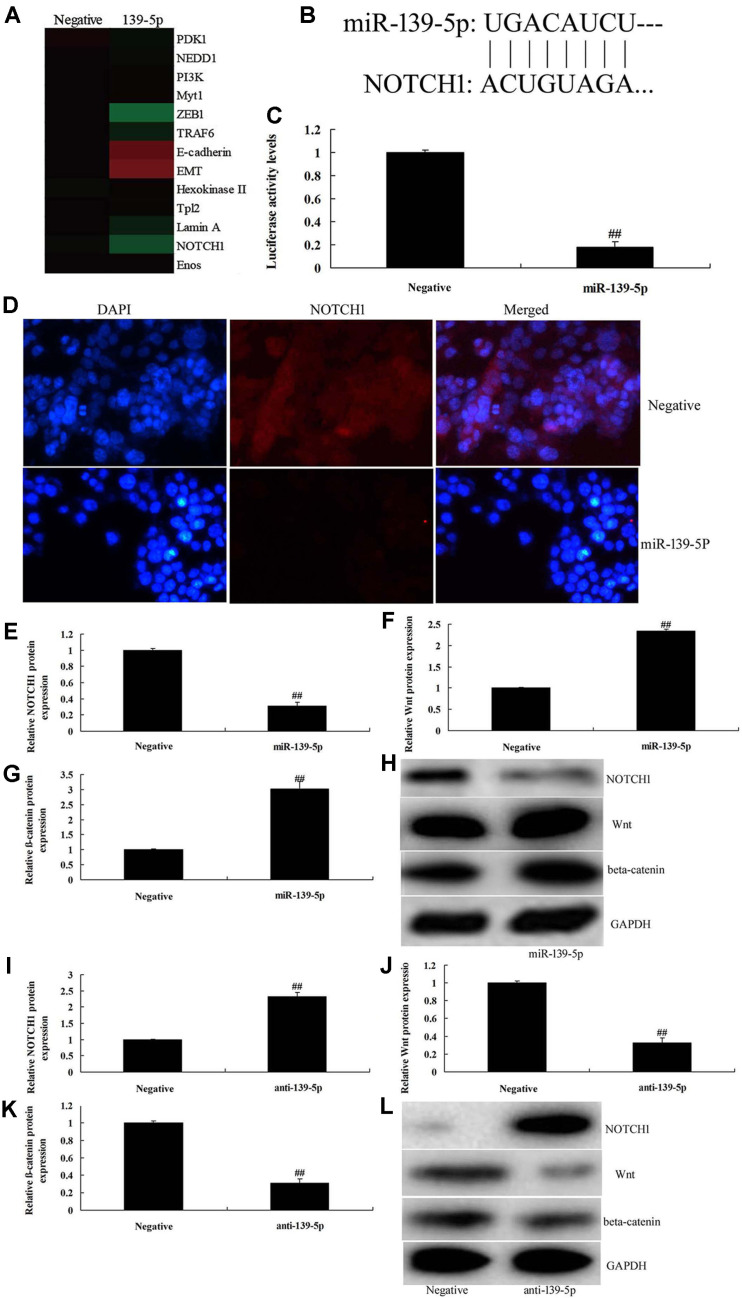
MicroRNA-139-5p regulates Wnt/β-catenin signaling pathway by NOTCH1 in BMSCs. Gene chip for signaling pathway (**A**), Notch1 is a direct target of microRNA-139-5p in BMSCs (**B**), and luciferase activity levels (**C**), Notch1 expression by IF (**D**), NOTCH1, Wnt and β-catenin expression using western blotting analysis (**E, F**, and **G**) and statistics analyzed (**H**) by overexpression of microRNA-139-5p; NOTCH1, Wnt and β-catenin expression using western blotting analysis (**I, J**, and **K**) and statistics analyzed (**L**) by down-reguation of microRNA-139-5p. Negative, negative mimics group; miR-139-5p, over-expression of miR-139-5p group; anti-139-5p, down-regulation of miR-139-5p group. ##*p* < 0.01 compared with negative mimics group.

We investigated the mechanism of microRNA-139-5p on osteogenic differentiation in BMSCs. As shown in Fig. 4A, over-expression of microRNA-139-5p reduced NOTCH1, and increased the Wnt/β-catenin signaling pathway in BMSCs, compared with the negative group. Notch1 is a direct target of microRNA-139-5p in BMSCs, and luciferase activity levels were inhibited in BMSCs by over-expression of microRNA-139-5p, compared with negative group (Figs. 4B and 4C). The results of IF showed that the over-expression of microRNA-139-5p suppressed NOTCH1 protein expression in BMSCs, compared with the negative group (Fig. 4D). Over-expression of microRNA-139-5p suppressed NOTCH1 protein expression, and induced Wnt and β-catenin protein expression in BMSCs, compared with the negative group (Figs. 4E-4H). Down-regulation of microRNA-139-5p induced NOTCH1 protein expression, and suppressed Wnt and β-catenin protein expression in BMSCs, compared with the negative group (Figs. 4I and 4J). Our results also showed that MicroRNA-139-5p regulates Wnt/β-catenin signaling pathway by NOTCH1 in BMSCs.

### The Inhibition of NOTCH1 Reduced the Effects of Anti-miR-139-5p on Cell Proliferation and Osteogenic Differentiation in BMSCs

**Fig. 5:**
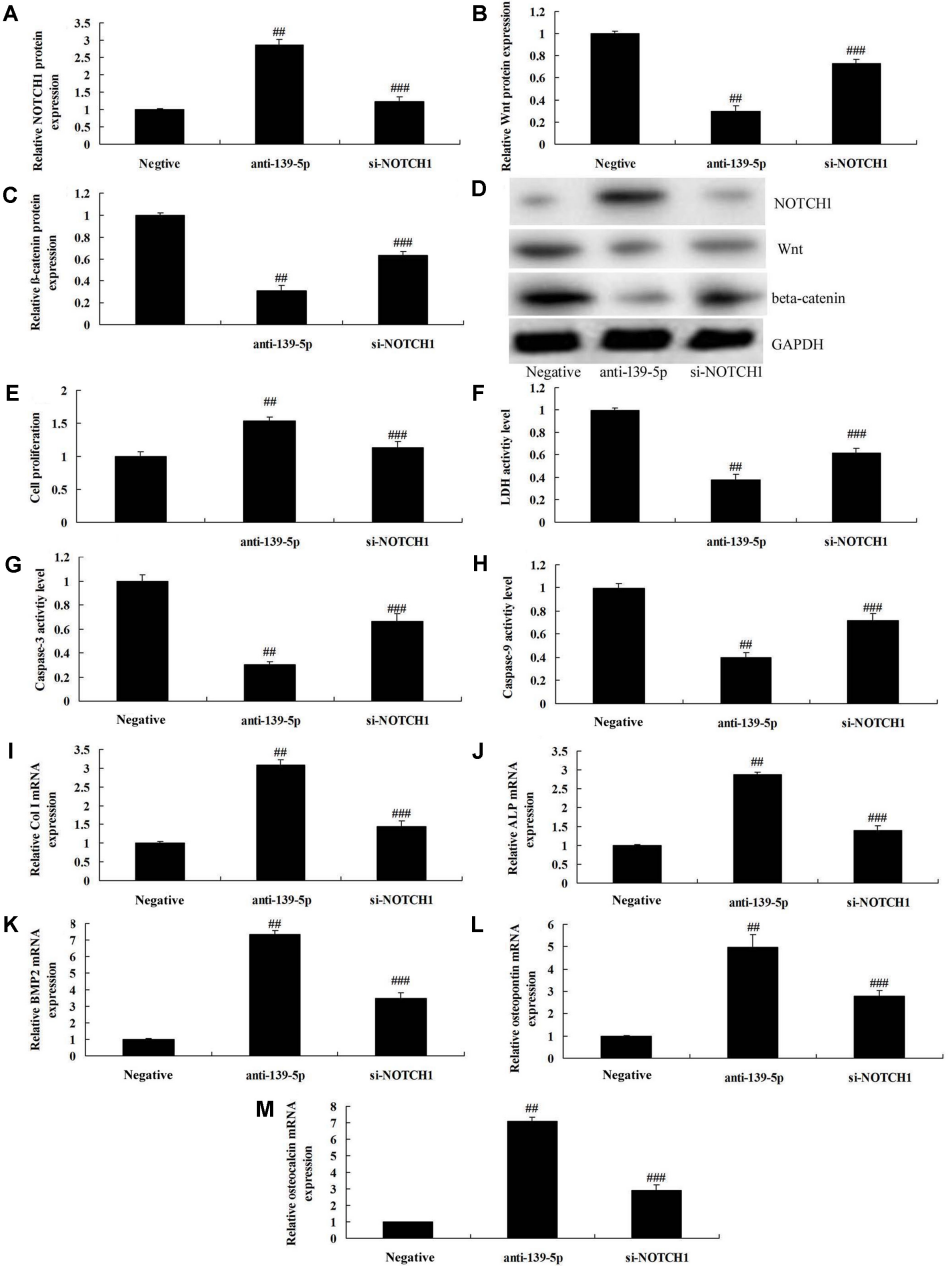
The inhibition of NOTCH1 reduced the effects of anti-miR-139-5p on cell proliferation and osteogenic differentiation in BMSCs. NOTCH1, Wnt and β-catenin expression using western blotting analysis (**A, B**, and **C**) and statistics analyzed (**D**), cell proliferation (**E**), LDH activity level (**F**) and caspase-3/9 activity levels (**G and H**), and Col I (**I**), ALP (**J**), BMP2 (**K**), osteopontin (**L**) and osteocalcin (**M**) mRNA expressions. Negative, negative mimics group; anti-139-5p, down-regulation of miR-139-5p group; si-NOTCH1, down-regulation of miR-139-5p and si-NOTCH1 group. ##*p* < 0.01 compared with negative mimics group, ###*p* < 0.01 compared with down-regulation of miR-139-5p group.

To confirm whether NOTCH1 participated in the effects of microRNA-139-5p on osteogenic differentiation of BMSCs, si-NOTCH1 was used to inhibit NOTCH1 expression. Si-NOTCH1 suppressed NOTCH1 protein expression, and induced Wnt and β-catenin protein expression in BMSCs by down-regulation of microRNA-139-5p, compared with down-regulation of the microRNA-139-5p group (Figs. 5A-5D). The inhibition of microRNA-139-5p reduced cell proliferation, increased LDH activity level and caspase-3/9 activity levels, and inhibited Col I, ALP, BMP2, osteopontin and osteocalcin mRNA expressions in BMSCs, compared with negative group (Figs. 5E-5M).

### Activation of Wnt/β-Catenin Also Inhibited the Effects of Anti-miR-139-5p on Cell Proliferation and Osteogenic Differentiation in BMSCs 

**Fig. 6:**
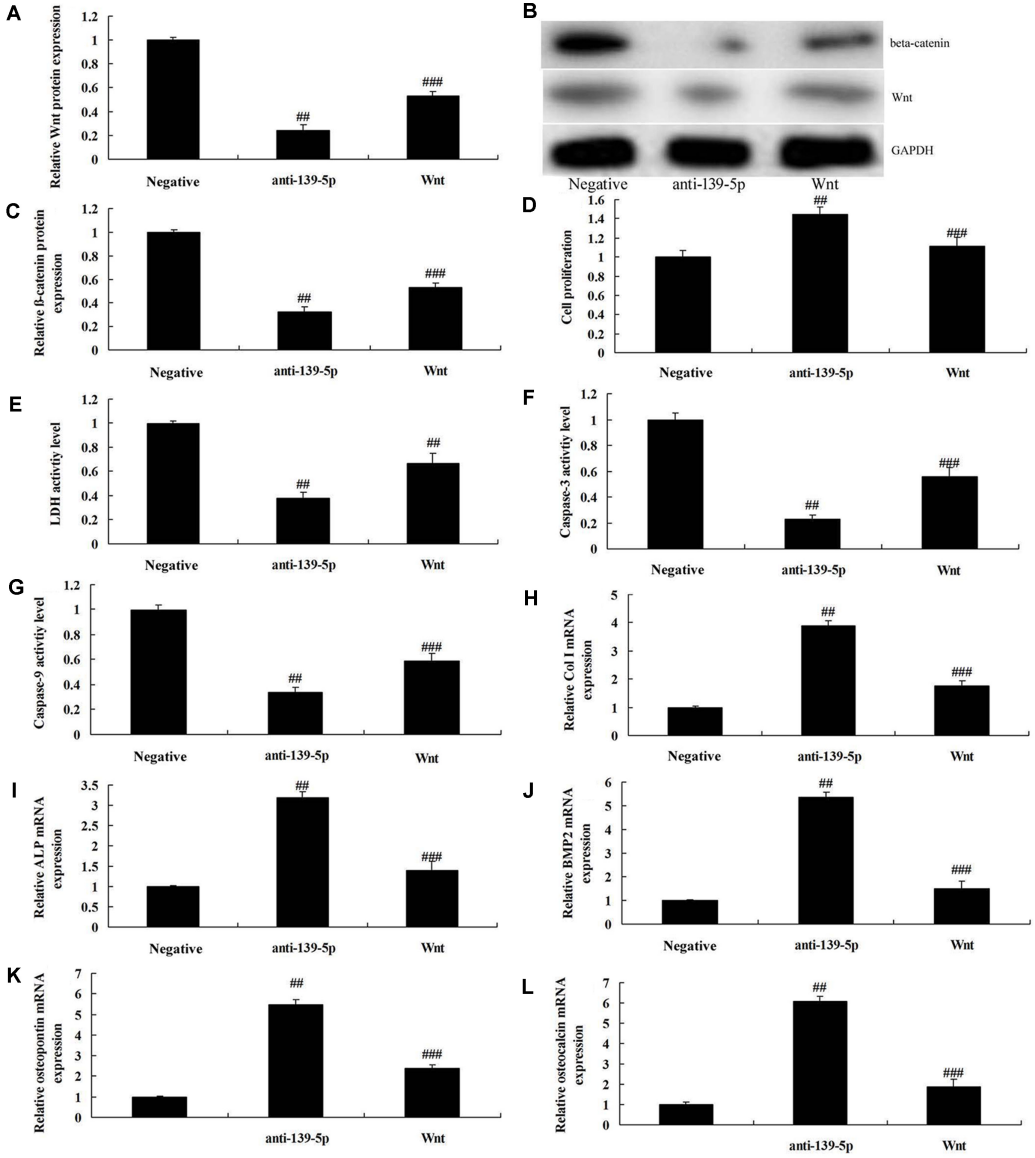
Activation of Wnt/β-catenin also inhibited the effects of anti-miR-139-5p on cell proliferation and osteogenic differentiation in BMSCs. Wnt and β-catenin expression using western blotting analysis (**A** and **B**) and statistics analyzed (**C**), cell proliferation (**D**), LDH activity level (**E**) and caspase-3/9 activity levels (**F** and **G**), and Col I (**H**), ALP (**I**), BMP2 (**J**), osteopontin (**K**) and osteocalcin (**L**) mRNA expressions. Negative, negative mimics group; anti-139-5p, down-regulation of miR-139-5p group; si-NOTCH1, down-regulation of miR-139-5p and si-NOTCH1 group. ##*p* < 0.01 compared with negative mimics group, ###*p* < 0.01 compared with down-regulation of miR-139-5p group.

We also studied whether the suppression of Wnt/β-catenin participated in the effects of microRNA-139-5p on osteogenic differentiation in BMSCs. Wnt plasmid induced Wnt and β-catenin protein expression in BMSCs by down-regulation of microRNA-139-5p, compared with down-regulation of the microRNA-139-5p group (Figs. 6A-6C). Activation of Wnt/β-catenin also inhibited the effects of anti-miR-139-5p on the inhibition of cell proliferation, the promotion of LDH activity level and caspase-3/9 activity levels, and the reduction Col I, ALP, BMP2, osteopontin and osteocalcin mRNA expressions in BMSCs, compared with the anti-miR-139-5p group (Figs. 6D-6L).

## Discussion

BMSCs, which derive from bone marrow, have self-renewing and multi-directional differentiation potential, and can differentiate into osteoblasts, chondrocytes, adipocytes and myocytes, respectively, under various induction conditions [13, 14]. At present, mesenchymal stem cells are frequently used as seed cell in bone regeneration research, but how to induce their directional osteogenic differentiation has always been the key and a difficult area of research [15]. We demonstrated that the expression of microRNA-139-5p in a model of osteoporosis was promoted. Li *et al*. suggested that LINC00152 may function as an oncogene in oral squamous cell carcinoma and could be a potential therapeutic target in patients with  this disease [16].

The osteogenic regulatory mechanisms of stem cells can be divided into intracellular and intercellular space regulatory mechanisms [17]. Of them, the intracellular regulation can also be divided into the translational level, post-translational level, transcriptional level and post-transcriptional level; and the intercellular regulation derives from the contact regulation of the adjacent cells or paracrine regulation [18]. miRNA regulation belongs to the transcriptional and post-transcriptional regulatory mechanism [19]. Our findings support that over-expression of microRNA-139-5p reduced cell proliferation, increased LDH activity level and caspase-3/9 activity levels, and inhibited Col I, ALP, BMP2, osteopontin and osteocalcin mRNA expressions in BMSCs. Zhang *et al*. suggested that 25-hydroxycholesterol promotes RANKL-induced osteoclastogenesis through miR-139-5p [20].

The recent research finds that NOTCH1 can also induce the directional osteogenic differentiation of MSCs, with the effect being remarkably stronger than the other members in the BMP family. The present study showed that the over-expression of microRNA-139-5p suppressed NOTCH1 protein expression, and induced Wnt and β-catenin protein expression in BMSCs. Li *et al*. reported that Notch1 by miR-139-5p inhibits glioma metastasis and epithelial-mesenchymal transition [21].

RUNX2 is the recognized osteogenesis-associated transcription factor in the downstream BMPs, which plays a vital role in the development of the skeletal system [22]. No osteogenesis can be seen in the cultured calvarial cells from mouse after the deletion of RUNX2, and still no osteogenesis can be seen after induction by BMP2, but the cartilage matrix can be seen, suggesting that RUNX2 is indispensable in the genesis and development of osteogenesis [23]. Our results also showed that over-expression of microRNA-139-5p suppressed NOTCH1 protein expression, and induced Wnt and β-catenin protein expression in BMSCs. These results showed that NOTCH1 plays a key role in the effects of microRNA-139-5p on differentiation of BMSCs. Xie *et al*. showed that miR-139-5p elevates skeletal myogenic differentiation of human adult dental pulp stem cells (ADSCs) through the Wnt/β-catenin signaling pathway [24].

BMP2 can regulate the expression of ALP through the Wnt signaling pathway, and it promotes the expression of ALP and osteogenic calcium deposition through the Wnt autocrine ring; in addition, the stable expression of β-catenin is required in the Wnt pathway in order to promote the expression of ALP [25]. β-catenin also plays an important role in the BMP2-induced ectopic osteogenesis [26]. β-catenin, which has no obvious effect when used alone, may serve as an insufficient activator in osteogenic differentiation, improving the sensitivity of other signaling pathways, such as the BMP signaling pathway [27]. Our study showed that the activation of Wnt/β-catenin also inhibited the effects of anti-miR-139-5p on cell proliferation and osteogenic differentiation in BMSCs. Based on our results, we found that the Wnt/β-catenin signaling pathway is an important pathway for the effects of microRNA-139-5p on differentiation of BMSCs.

In conclusion, our results indicate that microRNA-139-5p promotes osteogenic differentiation of BMSCs via Wnt/β-catenin signaling pathway by targeting NOTCH1, and moreover, that microRNA-139-5p may have potential clinical applications in osteogenic differentiation of BMSCs or osteoporosis.
